# Enjoyment and Affective Responses to Moderate and High-Intensity Exercise: A Randomized Controlled Trial in Individuals with Subsyndromal PTSD

**DOI:** 10.3390/sports12050138

**Published:** 2024-05-20

**Authors:** Daniel R. Greene, Angelia M. Holland-Winkler, Steven J. Petruzzello

**Affiliations:** 1 Department of Kinesiology, Augusta University, 3109 Wrightsboro Road, Augusta, GA 30909, USA; awinkler@augusta.edu; 2 Department of Kinesiology and Community Health, University of Illinois at Urbana-Champaign, 906 S. Goodwin Ave, Urbana, IL 61801, USA; petruzze@illinois.edu

**Keywords:** exercise, enjoyment, affective state, affective valence, energy, intensity, PTSD

## Abstract

This crossover randomized controlled trial examined the acute psychological effects of a bout of moderate-intensity continuous aerobic exercise (MICE) and a bout of high-intensity functional exercise (HIFE), relative to a no-exercise sedentary control (SED), in participants (N = 21; 15 f; 24.7 ± 9.3 years) with subsyndromal post-traumatic stress disorder (PTSD). Affective state (Energy, Tiredness, Tension, Calmness) was assessed before (Pre), immediately after (Post 0), 20-min after (Post 20), and 40-min after (Post 40) each condition. Affective valence was assessed during each condition, and exercise enjoyment was assessed at Post 0. Enjoyment was significantly greater following HIFE and MICE relative to SED. Energy was significantly increased Post 0 HIFE and MICE but decreased Post 0 SED. Tension was reduced following all conditions and was significantly lower at Post 40 relative to Pre for HIFE, MICE, and SED. Tiredness was significantly reduced at Post 40 relative to Pre following MICE only, while Calmness was significantly lower at Post 40 relative to Pre following MICE and SED. Overall, both exercise conditions were enjoyed to a greater extent than the control, but MICE may provide greater psychological benefits with respect to Calmness and Tiredness. This study is among the first to assess acute changes in affective states relative to various exercise modes in individuals living with subsyndromal PTSD.

## 1. Introduction

Post-traumatic stress disorder (PTSD) is a mental health condition brought on by one or more traumatic events and ranges in severity. The lifetime prevalence of PTSD in the United States has been estimated at 6.8% [1], however, other populations can experience elevated risk. Specifically, PTSD prevalence has been cited at 15% in combat veterans [2], and 26% of first responders report significant PTSD symptomology [3]. Psychological health conditions may arise due to PTSD such as anxiety, depression, and sleep disorders [4,5]. In addition to mental health, physical health is also often impaired. Physical impairments associated with PTSD include chronic pain [6], hypothalamic–pituitary–adrenal axis dysfunction [7], diabetes, hypertension, and hypercholesterolemia [8]. Those with PTSD are at an increased risk for cardiovascular disease, metabolic syndrome [9], and pulmonary disease [4,10]. Individuals with greater PTSD symptoms (i.e., number and/or severity) have an elevated risk for general health symptoms and medical conditions as well as poorer health-related quality of life [11,12]. Overall, those with PTSD have both psychological and physical health impairments that put them at an increased risk for early biological aging [13].

Given the health concerns associated with PTSD, both pharmacological and psychological interventions have been explored with optimistic results [14]. A recent meta-analysis identified positive effects for well-established, empirically supported psychological interventions to treat PTSD including cognitive therapy (g = 1.63), exposure therapy (g = 1.08), and eye movement desensitization and reprocessing [15]. Furthermore, common pharmacological interventions to treat PTSD have been associated with an effect size of 0.74 for paroxetine, 0.41 for sertraline, 0.43 for fluoxetine, 0.41 for risperidone, 1.20 for topiramate, and 0.48 for venlafaxine [15]. While these well-established, empirically supported treatments for PTSD are effective, there are some concerns regarding the participant retention and response rates. Treatment outcome data suggest dropout rates as high as 50% and nonresponse rates as high as 67% for exposure therapy, dropout rates as high as 32% and nonresponse rates as high as 71% for cognitive behavioral therapy, and dropout rates as high as 36% and nonresponse rates as high as 92 percent for eye movement desensitization and reprocessing [16]. A recent study on veterans diagnosed with PTSD and prescribed medication indicated a 34.6% dropout rate within 30 days and a 71.8% dropout rate within 180 days [17]. Other studies have highlighted nonresponse rates around 33% [18] and dropout rates between 19 and 27% [19] following empirically supported PTSD treatments. Additionally, psychological interventions often require one-on-one manual therapy by a highly trained therapist. Individuals may have limited access to this type of therapy due to the availability of therapists and insurance coverage [20]. This level of treatment response suggests that individuals with PTSD may benefit from a combination of treatment lines.

As above-mentioned, individuals living with PTSD experience an elevated risk for numerous health concerns and medical conditions. Regular physical activity provides physical and mental health benefits [21,22,23] that may reduce comorbid symptoms associated with PTSD. Specifically, physical activity improves blood pressure [24], hypothalamic–pituitary–adrenal axis function [23], and cholesterol [24,25] as well as reduces the risk for cardiovascular disease [26], metabolic syndrome [27], and pulmonary disease [28]. Physical activity also improves mental health conditions related to PTSD such as depression, anxiety, and sleep disorders. Following 3-weeks of regular exercise, individuals with multiple sclerosis displayed a significant reduction in depression, fatigue, and sleep complaints [29]. Numerous meta-analyses on the anxiolytic effects of exercise on anxiety have revealed moderate effect sizes [30,31,32], highlighting the beneficial effect of exercise on anxiety. However, disengagement resulting from a mental illness may cause these individuals to lead a more sedentary lifestyle despite the health benefits associated with physical activity [33]. While these studies were not conducted within a population living with PTSD, it follows that many of these benefits would translate to this special population. 

Applying exercise interventions for individuals with PTSD has the potential to reduce comorbid symptoms of PTSD and possibly reduce PTSD symptom severity. A recent systematic review and meta-analysis identified 11 studies assessing the exercise effects on PTSD. The results indicate that exercise has a beneficial effect (i.e., small to moderate effect sizes) on PTSD symptoms, depressive symptoms, sleep disturbances, and substance use disorder [4]. Another review identified exercise as an effective treatment to reduce PSTD symptomology in individuals with subsyndromal PTSD and highlighted the beneficial effects of comorbid symptoms such as anxiety, depression, and sleep disturbances [34]. Additionally, sport and game programs are commonly used as a strategy to reduce PTSD symptoms. However, in an attempt to systematically review randomly controlled trials that evaluated the effectiveness of sports and games on reducing PTSD symptoms, Lawrence, De Silva, and Henley [14] found no studies that met the criteria to be included in the review, thus there is a great need for randomized, controlled trials in this population. Rosenbaum, Sherrington, and Tiedemann [35] conducted a novel randomized controlled trial to help support this need. They found that patients diagnosed with PTSD in a 12-week exercise program that included a combination of resistance training and walking in addition to standard of care had significant reductions in PTSD symptoms and depressive symptoms compared to the control group that only received the standard of care [35]. This study is an example of how exercise can be used in conjunction with usual care, as participants received a combination of psychotherapy, pharmacology, and group therapy interventions during the study [35]. 

Although previous research has demonstrated the positive effects of physical activity on mental health including PTSD, there is still a need for randomized controlled trials that provide outcomes on the intensity and mode of physical activity that may increase exercise adherence in a population living with PTSD or PTSD symptoms. According to hedonic theory [36,37], individual behaviors are highly motivated by the pursuit of pleasure and avoidance of displeasure/pain. Additionally, the dual-mode theory states that in-task valance remains positive at exercise intensities below the ventilatory threshold (VT) or lactate threshold (LT), becomes variable at the VT or LT, and steadily declines at intensities above the VT or LT [38,39,40,41]. Therefore, exploring changes in affective states from pre-to-post exercise and examining in-task valance could provide valuable information leading to increased exercise adherence [42,43,44]. Furthermore, comparing post-exercise enjoyment may provide additional information on exercise adherence as enjoyment has been shown to increase exercise adherence and reduce dropout rates [45]. While previous work has highlighted the beneficial effects of exercise at low, moderate, and high intensity [46], physical activity rates remain a concern with about 54% of U.S. adults meeting aerobic physical activity guidelines and about 24% meeting both aerobic and resistance exercise guidelines [47]. 

While research assessing affective state changes, in-task valance, and post-exercise enjoyment in individuals living with PTSD and PTSD symptoms is limited, previous research has highlighted these effects in other clinical and healthy populations. Brand et al. [48] reported improved mood following the completion of 40–60 min of various modes of exercise (i.e., ball sports, Nordic walking, and workout/gymnastics) in inpatients with a variety of mental disorders. Meyer et al. [49] assessed changes in depressed mood via the Profile of Mood States [50] and brain-derived neurotrophic factor (BDNF) responses via blood draws following the completion of 30-min of aerobic cycling at low, moderate, high, and a preferred intensity in adult females with a major depressive disorder. Results suggest that the imposed exercise intensities were more advantageous for improving depressed mood and increasing BDNF responses. Greene et al. [51] examined changes in affective state, in-task valance, and enjoyment following 15-min of walking, quiet rest, and a high-intensity body weight interval exercise session in college students. Their results indicated an elevated enjoyment following both walking and body weight exercise conditions relative to quiet rest, increased in-task valance during walking, and decreased in-task valance during quiet rest and body weight exercise, and improved affective states following walking and body weight exercise relative to quiet rest [51]. Moreover, the study by Jung et al. assessed the changes in in-task valance and enjoyment during and following a single bout of high-intensity interval exercise (HIT), moderate-intensity continuous exercise (MICE), and vigorous-intensity continuous exercise (VICE) in healthy men and women. Participants completed one min at 100% Wpeak followed by one min at 20% Wpeak for 20 min during HIT, 40 min at 40% Wpeak during MICE, and 20 min at 80% Wpeak during VICE [52]. Results indicated greater enjoyment following HIT relative to MICE or VICE, and increased positive in-task valance during MICE relative to HIT and VICE [52]. As above-mentioned, exercise at high intensity (i.e., above VT or LT) typically results in a decrease in positive affect and an increase in negative affect, however, these responses are less understood under the context of interval exercise. A recent review has explored this phenomenon and concluded that enjoyment following interval exercise (often of higher intensity) is equal to or greater than enjoyment following continuous exercise [53]. 

Given the significant benefits exercise has shown on PTSD symptoms [35], comorbid symptoms of PTSD [29], and improved mood [48], nonadherence to exercise programs, especially within this population, warrants investigation. As above-mentioned, only 54% of adults meet the aerobic physical activity guidelines [47], with individuals suffering from a mental health condition at an increased risk of inactivity. A recent systematic review and meta-analysis indicated that individuals with PTSD were 9% less likely to be physically active and 31% more likely to be obese than the general population [54]. Furthermore, Assis et al. assessed the physical activity levels in individuals diagnosed with PTSD and concluded a significant decrease in activity following diagnosis. Specifically, the physical activity rates were about 52% in individuals without a PTSD diagnosis but dropped to 22% in individuals diagnosed with PTSD; participants identified time and lack of motivation as major reasons for nonadherence to exercise [33]. Individuals living with PTSD often avoid social interaction and may need alternatives to traditional gym-based exercise programs. A recent survey of men diagnosed with PTSD identified exercising at home and alone as two of the most attractive exercise options [55]. Additionally, Pebole et al. examined exercise preferences in 355 females with PTSD and reported 75% of participants preferred to exercise at home, 15% preferred a gym setting, and about 10% preferred exercising outside or associated with a medical provider. Furthermore, 75% of participants preferred to exercise alone or online, 23% preferred to exercise in a group, and about 1% indicated no preference [56]. As such, it may be beneficial to explore the acute changes in affective states, in-task valance, and the enjoyment of exercise that can be carried out at home, with minimal equipment and time investment. High-intensity interval exercise has been proposed as a time effective alternative to traditional cardio, providing similar if not enhanced physiological outcomes [57], but much less is known about the acute psychological outcomes. 

Therefore, the present study aimed to explore three main purposes. The first purpose was to assess the post-exercise enjoyment following high-intensity functional exercise (HIFE), moderate-intensity continuous exercise (MICE), and a sedentary, no-exercise period (SED). The second purpose was to examine the changes in affective states from before, immediately after, and 20 and 40 min after each condition. The third was to examine the in-task valence during each condition. Based on previous research that assessed enjoyment and affective responses to high-intensity interval and moderate-intensity aerobic exercise [51,52,53], it was hypothesized that post-exercise enjoyment would be similar following HIFE and MICE, but that both conditions would result in greater enjoyment relative to SED. Second, it was hypothesized that participants would report significant increases in positive affect and decreases in negative affect after the completion of HIFE and MICE relative to SED. Finally, it was hypothesized that in-task valance would be significantly more positive during MICE relative to HIFE.

## 2. Materials and Methods

### 2.1. Experimental Design

**Figure 1:**
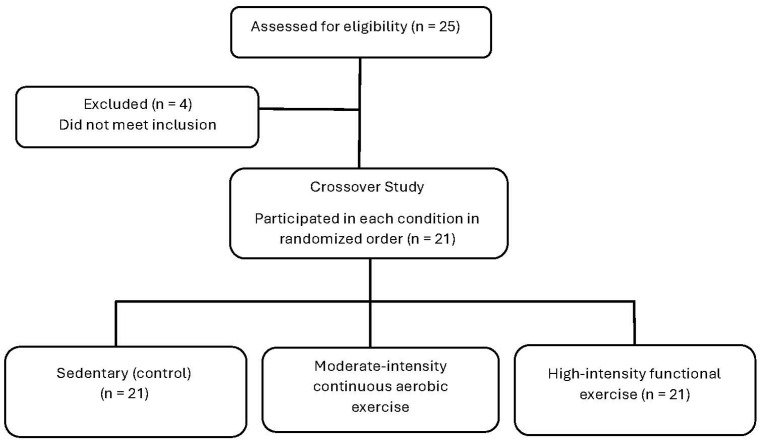
Flow diagram of participant recruitment.

A randomized, controlled, counterbalanced crossover design was used to compare different exercise intensities and modes on enjoyment and affective responses in participants with subsyndromal PTSD. The study consisted of four laboratory visits. The first visit included filling out the informed consent and health questionnaires in addition to measuring the VO2peak, which was used to determine the intensity levels for subsequent visits. The following three visits included exercise testing at one of three exercise intensities, in random order, with at least 48-h between visits (see Figure 1). All visits occurred in a temperature-controlled laboratory. This study was approved by the University’s Institutional Review Board, and all procedures followed the institutional guidelines.

### 2.2. Participants 

Participants with subsyndromal PTSD were recruited across a large college campus. A total of 25 participants were assessed, but only 21 met the including criteria for subsyndromal PTSD. Inclusion criteria were as follows: (1) ages 18–64; (2) self-reported exposure to a traumatic event (i.e., criterion A); (3) having at least one symptom for criterion B (i.e., re-experiencing), one symptom for criterion C (i.e., avoidance), two symptoms for criterion D (i.e., negative alterations in cognitions and mood), and two symptoms for criterion E (i.e., hyper-arousal: [58]); and (4) completion of the Physical Activity Readiness Questionnaire to determine if exercise was likely safe [59]. A symptom was defined when participants scored a 2 (i.e., moderately) or higher on the PCL-5 Likert scale. Furthermore, all participants signed the university approved informed consent document and no participant reported any contraindications to physical activity. About half of the participants (43%) reported exercising vigorously on a regular basis [exercise frequency = 3.7 ± 1.5 days per week, duration = 65.6 ± 27.9 min per session, intensity = 4.3 ± 1.1 (5 = hard, 7 = very hard using the CR-10 RPE scale; [60])]. The final sample identified as predominantly Caucasian (i.e., 85.7%); other descriptive information of the participant sample is included in Table 1. The participants were asked to abstain from exercising and consuming alcohol 24 h before each testing session. 

### 2.3. Sample Size Calculation

A post hoc calculation was conducted using prior work as a guide G*Power [61] to determine whether the sample size was sufficient. Based on previous results assessing changes in affective states following acute exercise [62], the following parameters were defined: Cohen’s f: 0.47; alpha error probability: 0.05; 1-beta: 0.95; with three conditions and four time points; and an estimated correlation among repeated measures of 0.5, therefore, the sample size needed to detect a significant effect was determined to be 15. As such, the 21 participants that met our inclusion criteria were deemed satisfactory. 

### 2.4. Visit 1

Following the completion of all of the initial questionnaires (i.e., informed consent, PCL-5, PAR-Q), the participants’ aerobic capacity was assessed. All participants completed a VO2peak test using a Bruce protocol. Starting at 1.7 mph and a 10% grade, participants completed 3-min stages until their heart rate reached ~85% of their age-predicted maximal heart rate (HR; calculated as 208 − 0.7 × age), a respiratory exchange ratio (RER) of 1.1, or the participant reached volitional exhaustion. After each completed stage, the grade was increased by 2% and speed increased to 2.5, 3.4, 4.2, 5.0, and 5.5 mph, respectively, with each stage [63]. All participants were able to achieve either an RER of 1.1 or age-predicted maximal heart rate of 85% before reaching volitional exhaustion. Following completion of the VO2peak test, participants were monitored until baseline HR was reached. 

### 2.5. Visits 2–4

Visits 2 through 4 were randomized and counterbalanced. All participants completed each of the three conditions: high-intensity functional exercise (HIFE), moderate-intensity continuous exercise (MICE), and a no exercise control (SED) condition. Each condition was 35 min, followed by a 40-min monitoring period (i.e., 75 min total). Affective states were assessed at pre (Pre), immediately post (Post 0), 20 min post (Post 20), and 40 min post (Post 40) each condition. Affective states were assessed at various time points to minimize the possibility of missing transient and/or delayed onset changes. Enjoyment was assessed at Post 0 only. In-task valence was assessed every 5-min during each condition, with in-task valence being assessed immediately after each 3-min of activity during HIFE. In-task valence was included to capture how individuals responded during exercise, as this can have a large impact on enjoyment and adherence to exercise [51,53]. Additionally, to maintain appropriate exercise intensities, HR (i.e., continuously monitored) and RPE (i.e., assessed every 5 min) were collected during each condition. For safety, there was a minimum of two research members present, and the participants were permitted to drink water ad libitum. All three conditions were completed within two weeks, with a minimum of 48 h between sessions to limit extraneous variables. 

### 2.6. HIFE

During HIFE, participants completed a 5 min warm-up, 25 min of active exercise, and a 5 min cool-down. Both the warm-up and cool-down were completed at 37–45% of their VO2peak (i.e., 57–63% HRmax), which is the recommended light intensity protocol for a warm-up and cool-down [64]. Active exercise consisted of five circuits conducted at a ratio of 3 min of activity followed by 2 min of rest. Activity involved completing three blocks of resistance exercises and two blocks of aerobic exercises. During each block, the participants completed three specific exercises/movements for 30 seconds and repeated each exercise/movement twice per block (see Table 2). During each active circuit, the participants were encouraged to work as hard as they could. The HIFE exercise protocol was created to provide a complete full-body workout that the participants could complete with minimal or no equipment in the comfort of their homes. Individuals with PTSD have indicated a strong preference for exercising both at home and alone [55,56]. Additionally, the American College of Sports Medicine recommends incorporating strength training 2 days per week for each major muscle group [65].

### 2.7. MICE

During MICE, the participants completed the same warm-up and cool-down as HIFE, but active exercise was 25 min of moderate-intensity aerobic exercise on a treadmill. Treadmill speed and grade were manipulated to keep the RPE between 12 and 15 (“somewhat hard” to “hard” [60]) and HR between 64 and 76% HRmax (i.e., 46–63% VO2peak [64]). To keep a valid assessment of RPE, speed and grade were manipulated following the administration of the RPE scale (i.e., every 5 min when necessary).

### 2.8. SED

During SED, the participants remained seated in the research lab. All interactions were kept identical to both the HIFE and MICE including the HR and RPE assessments.

### 2.9. Measures

#### 2.9.1. PTSD Checklist for DSM-5 (PCL-5)

The PCL-5 was used to assess the PTSD symptom severity [58]. Participants were instructed to report how much they had been bothered by each statement in the past month using a 5-point Likert scale (i.e., “Not at All” (0); “A Little Bit” (1); “Moderately” (2); “Quite a Bit” (3); “Extremely” (4)). While the gold standard for diagnosing PTSD is a structured clinical interview, the PCL-5 can provide a provisional PTSD diagnosis and has demonstrated strong internal consistency (α = 0.94) and test–retest reliability (r = 0.82; [66]).

#### 2.9.2. The Physical Activity Enjoyment Scale (PACES)

Post-exercise enjoyment was measured with the PACES [67]. This 18-item self-report measure has demonstrated strong internal consistency (α = 0.93). Participants were instructed to pick the number that most closely matched how they felt about the activity they had just completed by using a 7-point Likert scale (e.g., “It’s no fun at all” (1) to “It’s a lot of fun”(7)). PACES scores range from 18 to 126, with scores for the present study ranging from 50 to 126, 69 to 126, and 42 to 120 following the HIFE, MICE, and the SED conditions, respectively. 

#### 2.9.3. The Activation Deactivation Adjective Checklist (AD ACL)

Affective states were assessed using the AD ACL [68]. The AD ACL is a 20-item self-report measure that provides a valid assessment for four subscales: Energy, Tiredness, Calmness, and Tension. Participants were instructed to indicate how they felt right now for each key word (e.g., “Active”, “Still”, etc.) on a 4-point Likert scale (i.e., “Not at All” (1); “Somewhat” (2); “Moderately So” (3); or “Very Much So” (4)). The AD ACL captures the major quadrants of the rotated affective circumplex, which include activated–pleasant, activated–unpleasant, unactivated–pleasant, and unactivated–unpleasant [69]. Scores for the AD ACL range from 5 to 20. 

#### 2.9.4. Feeling Scale (FS)

In-task valence was assessed using the FS [70]. The FS is an 11-point, single-item, bipolar measure of pleasure–displeasure. Participants were instructed to indicate how they felt right now on a scale that ranged from +5 to −5, with an option for neutral. Keywords were provided for +5 (i.e., “Very Good”), −5 (i.e., “Very Bad”), 0 (i.e., Neutral), and at every odd integer. 

#### 2.9.5. Rating of Perceived Exertion (RPE)

Perceptions of effort were assessed with the 15-point RPE scale [60]. The RPE is a self-report measure of effort that ranges from 6 (no exertion at all) to 20 (maximal exertion). The RPE was used during each condition, and the participants indicated how hard they felt they were working right now. The RPE scale has been validated in the literature as an appropriate method to assess exertion within an exercise setting (r = 0.884; [71]).

#### 2.9.6. Heart Rate (HR)

Participants were fitted with a Polar© FT1 HR monitor and Polar WearLink Coded 31 transmitters (Polar Electro, Kempele, Finland). HR values were continuously monitored during all conditions. 

### 2.10. Data Analysis

Data analysis was conducted using SPSS 27.0.0 for Windows. Data were initially inspected for any unusual data points. As none were found, all participants were included in all analyses. The analysis of differences in the main outcome variable for enjoyment was conducted with a Condition [3: HIFE, MICE, SED] by Time [1: Post 0] one-way analyses of variance [ANOVA]. Analysis of differences in the main outcome variables for Energy, Tiredness, Calmness, and Tension were conducted using a Condition [3: HIFE, MICE, SED] by Time [4: Pre, Post 0, Post 20, Post 40] repeated measure ANOVA, using a Bonferroni correction to protect against multiple comparisons. Finally, the analysis of differences in the main outcome variable for affective valence was conducted using a Condition [3: HIFE, MICE, SED] by Time [5: 5, 10, 15, 20, 25] repeated measure ANOVA, using a Bonferroni correction to protect against multiple comparisons. Effect sizes (ESs) were calculated with Cohen’s d ([72]: Cohen’s d = (M2 − M1)/SDpooled). 

## 3. Results

### 3.1. Enjoyment

**Figure 2:**
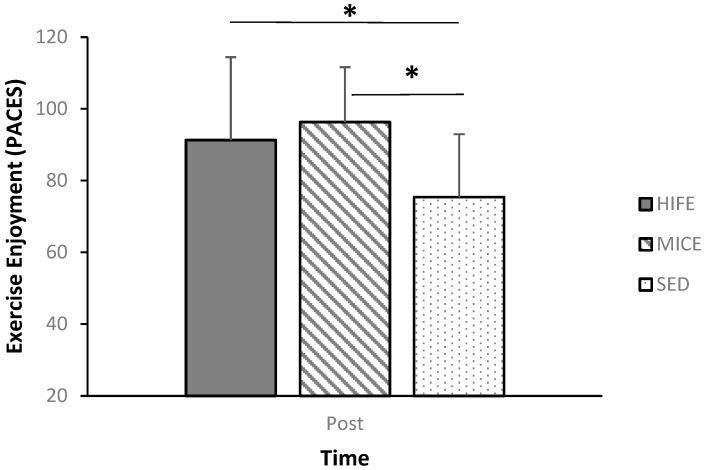
Post-condition enjoyment. * Indicates a significant difference at the *p* = 0.05 level.

Post-exercise enjoyment was assessed immediately after each condition. Participants indicated scores of 91.3 ± 23.1 (M ± SD), 96.3 ± 15.3 (M ± SD), and 75.4 ± 17.5 (M ± SD) following the HIFE, MICE, and SED conditions, respectively. Enjoyment was significantly greater following both HIFE [Mdiff ± SE; 15.9 ± 6.2; 95% CI: 0.3–32.1; *p* = 0.05; Cohen’s d = 0.78] and MICE [Mdiff ± SE; 20.9 ± 4.7; 95% CI: 8.7–33.2; *p* < 0.001; Cohen’s d = 1.27] relative to SED. Additionally, enjoyment was not different following HIFE [*p* = 0.85; Cohen’s d = 0.26] relative to MICE; see Figure 2. 

### 3.2. Affective State

Pre-to-post affective state changes in Energy, Tiredness, Tension, and Calmness were assessed using a RM ANOVA [Condition (3: HIFE, MICE, SED) by Time (4: Pre, Post 0, Post 20, Post 40)]. The RM ANOVA revealed a significant Condition effect for Energy [*p* = 0.002], Tiredness [*p* = 0.015], and Calmness [*p* = 0.049], but not for Tension [*p* = 0.364]. Additionally, there was a significant Time main effect for Energy [*p* < 0.001], Calmness [*p* = 0.003], and Tension [*p* < 0.001], but not Tiredness [*p* = 0.196]. As such, the Condition and Time main effects are examined below, and all Condition-by-Time interaction effects are highlighted in Table 2 and Table 3. 

Energy was significantly greater throughout HIFE [*p* = 0.027; Cohen’s d = 0.69] and MICE [*p* = 0.004; Cohen’s d = 0.67] relative to SED, while Tiredness was significantly less throughout MICE [*p* < 0.001; Cohen’s d = 0.63] relative to SED. Furthermore, Energy increased from Pre to Post 0 [*p* = 0.04; Cohen’s d = 0.57], and significantly decreased from Post 0 to Post 20 [*p* = 0.005; Cohen’s d = 0.47] and Post 40 [*p* = 0.001; Cohen’s d = 0.73]. Tiredness was significantly less throughout MICE [*p* < 0.001; Cohen’s d = 0.63] relative to SED [see Table 2 for all Condition × Time interaction effects].

The Condition main effect for Calmness [*p* = 0.049] was significant, but pairwise comparisons did not yield significant differences between any conditions. Calmness was significantly greater at Post 40 relative to Pre [*p* = 0.05; Cohen’s d = 0.66], while Tension was significantly reduced at Post 20 [*p* = 0.002; Cohen’s d = 1.05] and Post 40 [*p* < 0.001; Cohen’s d = 1.21] relative to Pre. Additionally, Tension was reduced at Post 40 relative to Post 0 [*p* = 0.005; Cohen’s d = 0.64]; see Table 4 for all Condition × Time interaction effects].

### 3.3. In-Task Valence

**Figure 3:**
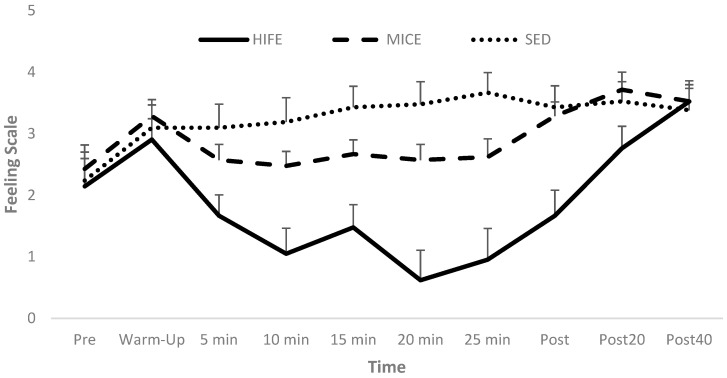
In-task affective valence.

The RM ANOVA for in-task valence revealed a significant Condition [*p* < 0.001] and Condition-by-Time interaction effect [*p* < 0.001], but the Time main effect was not significant [*p* = 0.220]. Specifically, in-task affective valence was more positive during both MICE [Mdiff ± SE; 1.4 ± 0.37; 95% CI: 0.47–2.4; *p* = 0.003; Cohen’s d = 0.99] and SED [Mdiff ± SE; 2.2 ± 0.55; 95% CI: 0.77–3.7; *p* = 0.002; Cohen’s d = 1.31] relative to HIFE, with no difference between MICE and SED [*p* = 0.157]. Additionally, the affective valence was not different between conditions at Pre [*p* = 0.76] or at Post 40 [*p* = 0.93; see Figure 3]. 

### 3.4. Manipulation Check

To determine whether participants met the appropriate exercise intensity for HIFE, MICE, and SED, the RPE and HR were assessed during each condition. The Condition main effect was significant for both HR and RPE. RPE was greater during HIFE [M ± SE; 14.02 ± 0.25] than MICE [M ± SE; 11.48 ± 0.32] and SED [M ± SE; 6.41 ± 0.18]. Specifically, RPE was significantly greater during HIFE [Mdiff ± SE; 7.61 ± 0.27; 95% CI: 6.92–8.31; *p* < 0.001; Cohen’s d = 7.81] and MICE [Mdiff ± SE; 5.07 ± 0.34; 95% CI: 4.18–6.00; *p* < 0.001; Cohen’s d = 4.29] relative to SED. Furthermore, RPE was greater during HIFE [Mdiff ± SE; 2.54 ± 0.35; 95% CI: 1.63–3.45; *p* < 0.001; Cohen’s d = 1.94] relative to MICE. 

The average HR (bpm) during the active 3-min of HIFE [M ± SE; 167.60 ± 2.10] was greater than the average HR during MICE [M ± SE; 147.85 ± 1.41] and SED [M ± SE; 80.26 ± 2.36]. Specifically, HR during the active 3-min of HIFE was significantly greater relative to both MICE [Mdiff ± SE; 19.74 ± 2.17; 95% CI: 14.06–25.42; *p* < 0.001; Cohen’s d = 2.41] and SED [Mdiff ± SE; 87.34 ± 2.77; 95% CI: 80.12–94.55; *p* < 0.001; Cohen’s d = 8.53]. The total average HR was not different during HIFE when including the 2-min rest periods relative to MICE [*p* = 1.00], but both HIFE [Mdiff ± SE; 67.69 ± 2.29; 95% CI: 61.71–73.67; *p* < 0.001; Cohen’s d = 6.93] and MICE [Mdiff ± SE; 67.60 ± 2.56; 95% CI: 60.90–74.29; *p* < 0.001; Cohen’s d = 7.59] resulted in greater HR relative to SED.

## 4. Discussion

This randomized, counterbalanced crossover, controlled trial was designed to examine the effects of 25-min bouts of MICE and HIFE, relative to SED, on acute enjoyment and affective responses in individuals living with subsyndromal PTSD. Overall, this study demonstrated that acute bouts of both MICE and HIFE resulted in greater levels of enjoyment than the SED condition. MICE also reduced feelings of tiredness and increased feelings of calmness compared to the SED condition. This suggests that both bouts of MICE and HIFE may be useful approaches to improve acute psychological well-being in this population.

The first purpose of the present manuscript was to assess changes in post-exercise enjoyment. It was hypothesized that enjoyment would be greater following HIFE and MICE relative to SED. Both the moderate- and high-intensity exercise conditions yielded significantly higher scores on the PACES relative to the no-exercise control. Furthermore, enjoyment was not different following HIFE relative to MICE. The results of the present study support the hypothesis and agree with previous research assessing enjoyment following low-moderate intensity continuous, high-intensity interval exercise, relative to a no-exercise condition [51]. In a scoping review of the literature, Stork et al. [53] found that most studies reported exercise enjoyment to be similar or greater after high-intensity interval exercise compared to moderate-intensity continuous exercise. Furthermore, the review found that most of the participants in the cited studies preferred high-intensity interval exercise over moderate-intensity continuous exercise. As exercise enjoyment has been identified as a strong indicator for continued exercise participation [42,43,44], it could be a valuable tool to increase exercise adherence. 

It was also hypothesized that participants would show an increase in positive affective states and a decrease in negative affective states following the completion of both exercise conditions. To assess the pleasure and displeasure of HIFE and MICE, participants completed the AD ACL immediately before, immediately after, 20-min post, and 40-min post each condition. Given the nature of the AD ACL, measures of pleasure are highlighted by increases in pleasant–activated affective states (i.e., Energy) and/or pleasant–deactivated affective states (i.e., Calmness). Similarly, measures of displeasure are noted by increases in unpleasant–activated affective states (i.e., Tension) and/or unpleasant–deactivated affective states (i.e., Tiredness). The results generally support this hypothesis. Specifically, the participants indicated significantly higher levels of Energy from pre- to immediate post-exercise for both the HIFE and MICE conditions, while the participants reported a decrease in Energy from the pre- to immediate post-SED condition. Calmness was significantly reduced from pre- to 40-min post-MICE and SED, but was not different from pre- at any time following HIFE. Furthermore, the participants indicated a decrease in Tiredness for MICE relative to SED, whereas the HIFE condition did not differ from either the MICE or SED conditions. Tiredness was significantly decreased from pre- to immediately post-, 20-min post-, and 40-min post-MICE only. Tiredness remained unchanged during HIFE and SED at all time points relative to pre-. There were no differences between conditions for the unpleasant–activated state of Tension. 

Overall, the results for affective state changes agreed with the previous literature. Greene et al. [51] found increased energy and decreased tiredness following 15 min of walking and body weight interval exercise relative to a quiet rest condition. A systematic review and meta-analysis reported a main effect of 0.47 for increased energy following acute exercise [62]. Finally, Jung et al. [52] reported increased energetic arousal following moderate-intensity continuous exercise and an increase in tension arousal following high-intensity interval exercise and moderate-intensity continuous exercise, but a larger increase following high-intensity interval exercise. Human behavior is often motivated by the pursuit of pleasure and the avoidance of displeasure (hedonic theory: [37]). Participants felt more energy after moderate- and high-intensity exercise relative to not exercising at all. Moderate-intensity exercise also decreased feelings of tiredness and increased calmness. While both exercise conditions resulted in increased affective states relative to no exercise, there appears to be some added benefits to moderate-intensity exercise with respect to calmness and tiredness. 

Finally, it was hypothesized that in-task valance would be significantly more positive during MICE relative to HIFE. The results of the present study support this hypothesis, as in-task valence (i.e., feeling scale) was significantly more positive during MICE relative to HIFE. Previous literature has linked exercise intensity to in-task responses. Specifically, the higher the intensity, the less positive/more negative the in-task valence [42,43,44]. This is important as in-task valance has been shown to predict post-exercise enjoyment [51]. However, while the present study showed a significantly more positive in-task valance during MICE, the in-task valance remained positive during HIFE, and post-exercise enjoyment was not different between MICE and HIFE. This has practical implications on exercise adherence, as affective responses to interval exercise may not follow affective responses to traditional continuous exercise. Previous literature has highlighted an increase in negative affective responses to high-intensity exercise [41,73], possibly due to increases in physiological stressors such as lactate accumulation and increased respiration due to changes in oxygen and carbon dioxide levels, which stimulate the brain to elicit negative affective responses [41]. It is suggested that these negative affective responses from high-intensity exercise may reduce exercise adherence [44,74]. While the present study reported more positive in-task valence during MICE, in-task valence during HIFE remained positive, indicating that affective responses during interval exercise may not follow well-established patterns associated with continuous exercise. 

The present study is novel as findings on exercise enjoyment, affective states, and in-task valence from various exercise intensities in a population living with PTSD/PTSD symptoms in controlled, randomized trials are limited and greatly needed. Teixeira et al. [75] explored exercise affect in healthy young adults in a randomized controlled trial. Randomized controlled trials reduce bias and allow for a more accurate analysis of specific treatment effects [76]. The trial may consist of a parallel design (i.e., receives an assigned treatment or condition throughout the study) or crossover design (i.e., receives all treatments or conditions in a randomized fashion). Our study utilized a crossover design that is valuable as the participants serve as their own controls, thereby reducing interindividual variability [77]. To avoid carryover effects from the acute bouts of exercise, a wash-out period of at least 48 h was incorporated between sessions. The exercise treatment order was randomized for each participant to further reduce the carryover effects from the previous treatment in the overall results [76].

With regard to assessing the affective responses to high-intensity interval exercise relative to continuous exercise, significant attention needs to be given to the exercise intensity itself. While a considerable body of literature on continuous exercise suggests affective responses to exercise are generally pleasant at intensities below the ventilatory or lactate threshold, highly variable at the lactate or ventilatory threshold, and become less pleasant/more negative at intensities above the lactate or ventilatory threshold [38,39,40,41], much less is known about affective responses to interval exercise. Studies have shown the in-task valence to be less positive/more negative during high-intensity interval exercise relative to moderate-intensity continuous exercise [51,52], and enjoyment to be similar or higher following high-intensity interval exercise [53]. However, longitudinal evidence linking affective responses during/after interval exercise to adherence should be considered. A recent systematic search identified eight studies that reported physical activity levels at least 12 months following high-intensity interval versus moderate-intensity continuous exercise. The results highlight a substantial decrease in performed exercise intensity during high-intensity interval exercise that was unsupervised, and ultimately led to the conclusion that high-intensity interval exercise was not advantageous for adherence [78]. Thus, high-intensity interval exercise may not be sustainable at imposed exercise intensities. 

Furthermore, one of the confounding variables within the literature assessing high-intensity interval exercise is the operational definition used in various studies. Specifically, the work rest ratios and relative exercise intensities can vary drastically between studies. Additionally, it is difficult to classify interval exercise as high-intensity, vigorous-intensity, or moderate-intensity, as rest periods make reaching a physiological steady state impossible. As such, recent evidence has suggested using a percentage of peak workloads to assess the intensity during interval exercise [79]. With regard to rest periods, Martinez et al. reported greater feelings of pleasure during, and higher enjoyment following interval exercise using shorter intervals after controlling for total work [80]. Additionally, a recent study reported greater feelings of pleasure following resistance exercise that decreased intensity during exercise relative to resistance exercise that increased intensity during exercise [81], thus indicating that exercise intensity progression can impact affective responses. As high-intensity interval exercise was listed as the second most popular fitness trend in 2020 [82], there is a need to study affective responses to high-intensity interval exercise, but caution needs to be given when assessing the true exercise intensity and adherence to these programs. 

In the present study, exercise intensity was assessed using subjective and objective measures. First, the participants’ aerobic capacity was assessed via a VO2peak test. During MICE, their heart rate was monitored and maintained at 64–76% HRmax (i.e., 46–63% VO2peak). During HIFE, the intensity was not specifically controlled for, but the participants were instructed to give their “all out” effort. The VO2peak was reported instead of the VO2max as the criteria to meet VO2max was not met by all participants. Therefore, the true maximum heart rate was likely not reached, resulting in slightly elevated intensity percentages per condition. The average heart rate achieved during the active intervals of HIFE and average heart rate during MICE were 88% and 78% HRmax achieved during the VO2peak test, respectively. Using age-predicted maximal heart rate (i.e., 220-age), the participants achieved an average intensity level of 75.8% during MICE and 85.9% during HIFE [83,84]. The American College of Sports Medicine defines moderate intensity as 64–76% of maximum heart rate and vigorous intensity as 77–95% of maximum heart rate [64]. Therefore, with an increased maximum heart rate from a true VO2max test, and in agreement with intensity based on the age-predicted maximal heart rate, the HIFE condition and MICE condition meet the standards for vigorous and moderate intensity, respectively. Furthermore, subjective measures of intensity were assessed via the RPE scale. Immediately following the active intervals of HIFE, the average RPE was 14 (“somewhat hard to hard”, which indicates vigorous intensity), the average RPE during MICE was 11.5 (“fairly light to somewhat hard”, which is on the borderline of light to moderate intensity), and the average RPE during SED was 6.4 (“very, very light”, which indicates a sedentary control [64]).

There are several limitations to the present manuscript. First, as the present sample was a convenience sample, previous exercise history, sex, and ethnicity were not controlled for. While it would be of value to examine the affective responses and enjoyment in both regular exercisers and sedentary individuals, this was less of a concern for the present study as exercise history did not change the results when used as a covariate. Additionally, the present study included fifteen females and only six males; this gender imbalance was similar to the participant make-up in the repeated measures, randomized, and counter-balanced study by Jung et al. [52] examining high- and moderate-intensity exercise bouts on affective responses in healthy men and women. Thus, future studies should explore affective responses to exercise in a more balanced group of men and women or by comparing larger groups of men and women, especially in participants living with PTSD/PTSD symptoms, as women have been shown to have PTSD prevalence rates nearly double that of men [85]. Furthermore, the participants were not clinically diagnosed with PTSD prior to participation in the study. While this limits the reach of the present manuscript, this was less of a concern as the overall PCL-5 score was well above the recommended cut point for a probable PTSD diagnosis. According to the PCL-5 questionnaire, a validated measure of PTSD symptoms, a score between 33 and 80 suggests that the PTSD severity is above the clinical threshold [58,86]. The mean score for the 21 participants in the present study was 52.5 ± 12.2. While a PTSD diagnosis was not provided by a clinical psychologist, the results from the PCL-5 serve to verify the severity of PTSD symptoms of the participants. Therefore, it is likely that the present results would extend to include those with a clinical diagnosis, but further research would be needed to confirm this. Finally, it is important to note that symptoms of PTSD include avoidance and decreased mental and physical health. It is possible that the participants in the present sample are not representative of all individuals living with PTSD/PTSD symptoms, thus limiting the generalizability of the findings. However, it is feasible that individuals with greater health impairments would experience more significant improvements following exercise. Future studies are needed on clinical populations living with PTSD and other comorbidities. 

## 5. Conclusions 

In conclusion, this crossover study demonstrated that an acute bout of both moderate-intensity continuous exercise and high-intensity interval-based exercise resulted in increased enjoyment with positive outcomes on affective states compared to a sedentary control in individuals with subsyndromal PTSD. Energy was increased in both HIFE and MICE compared to SED, while MICE also resulted in greater psychological benefits concerning Calmness and Tiredness. In-task valance was more positive during MICE relative to HIFE, but in-task valence remained positive during HIFE. Overall, an acute bout of MICE and HIFE were well-tolerated in this special population and both resulted in immediate psychological benefits. While future explorations need to address some of the study limitations and expand these findings to longitudinal effects on affective states and enjoyment, the present manuscript provides preliminary evidence that exercise may be used as an additional treatment line for individuals living with PTSD/PSTD symptoms. Furthermore, it is of value to continue to determine the most enjoyable form of exercise that may lead to high adherence rates in order to improve both the physical and mental health of those suffering from PTSD symptoms.

## Figures and Tables

**Table 1:** Descriptive information of the sample participants.

Variable	All (*N* = 21)		Females (*n* = 15)		Males (*n* = 6)		Sex Differences	
	M	SD	M	SD	M	SD	M_diff_	Sig
Age (years)	24.7	9.3	26.3	10.4	20.5	3.8	5.8	0.201
Height (cm)	167.0	7.0	164.1	5.7	174.4	3.5	−10.3	<0.001
Body mass (kg)	69.4	16.1	68.2	18.5	72.3	7.6	−4.1	0.611
BMI (kg·m^−2^)	24.8	5.3	25.2	6.2	23.7	2.1	1.5	0.587
VO2peak (mL·kg^−1^·min^−1^)	33.4	9.8	28.7	7.1	45.0	4.4	−16.3	<0.001
PCL-5 score	52.5	12.2	54.3	12.2	48.2	12.1	6.1	0.311

**Table 2:** Full HIFE exercise protocol.

Exercises	Block 1	Block 2	Block 3	Block 4	Block 5
	Resistance	Cardio	Resistance	Cardio	Resistance
Exercises 1 and 4	Push-ups (bw)	Mountain climbers (bw)	Bent Over Rows (bb)	Burpees (bw)	Squats (bb)
Exercises 2 and 5	Triceps Extension (bb)	Jump Rope (bw)	Biceps Curl (bb)	Calf Jumps (bw)	Upright Row (bb)
Exercises 3 and 6	Shoulder Press (bb)	Butt Kicks (bw)	Army Rowers (bw)	High Knees (bw)	Elbow Plank (bw)

bb = body bars; bw = body weight; Each exercise performed for 30 s and repeated twice per block (i.e., 3 min blocks).

**Table 3:** Mean ± SD and effect sizes of Energy and Tiredness before and after each condition.

Measure	Condition	Time	M	SD	Cohen’s d	
					Pre-Post 0 Pre-Post 20 Pre-Post 40	Post 0–Post 20 Post 0–Post 40 Post 20–Post 40
Energy	HIFE	Pre	10.14	2.82	+0.89 *	−0.70 *
		Post 0	12.76	3.06	+0.16	−0.96 *
		Post 20	10.62	3.07	+0.08	−0.24
		Post 40	9.90	2.88		
	MICE	Pre	10.00	3.24	+0.84 *	−0.41 *
		Post 0	12.67	3.10	+0.35 *	−0.85 *
		Post 20	11.24	3.88	0.00	−0.35 *
		Post 40	10.00	3.21		
	SED	Pre	10.00	3.13	−0.45 *	+0.02
		Post 0	8.67	2.78	−0.42 *	+0.05
		Post 20	8.71	3.04	−0.37 *	+0.03
		Post 40	8.81	3.30		
Tiredness	HIFE	Pre	11.67	3.64	+0.19	−0.13
		Post 0	11.05	2.84	+0.07	−0.03
		Post 20	11.43	2.89	+0.16	+0.10
		Post 40	11.14	2.82		
	MICE	Pre	11.71	3.95	+0.63 *	−0.02
		Post 0	9.62	2.60	+0.57 *	−0.14
		Post 20	9.67	3.18	+0.44 *	−0.11
		Post 40	10.05	3.53		
	SED	Pre	12.14	3.82	+0.10	−0.28
		Post 0	11.81	2.77	−0.15	+0.02
		Post 20	12.67	3.28	+0.11	+0.28 *
		Post 40	11.76	3.08		

Note: Direction of effect size indicates positive (i.e., +) or negative (i.e., −) change in affective state. * indicates significant change at the *p* = 0.05 level.

**Table 4:** Mean ± SD and effect sizes calmness and tension before and after each condition.

Measure	Condition	Time	M	SD	Cohen’s d	
					Pre-Post 0 Pre-Post 20 Pre-Post 40	Post 0–Post 20 Post 0–Post 40 Post 20–Post 40
Calmness	HIFE	Pre	9.48	3.27	−0.13	+0.71 *
		Post 0	9.10	2.77	+0.52	+0.68 *
		Post 20	11.05	2.77	+0.51	+0.02
		Post 40	11.10	3.11		
	MICE	Pre	10.10	2.41	+0.06	+0.27
		Post 0	10.24	2.68	+0.34	+0.51 *
		Post 20	11.00	2.95	+0.58 *	+0.24
		Post 40	11.71	3.10		
	SED	Pre	10.14	2.92	+0.60 *	−0.04
		Post 0	12.05	3.41	+0.60 *	−0.15
		Post 20	11.90	2.98	+0.48 *	−0.11
		Post 40	11.57	3.01		
Tension	HIFE	Pre	8.57	2.73	+0.16	+0.79 *
		Post 0	8.19	1.99	+0.81 *	+0.94 *
		Post 20	6.76	1.61	+0.93 *	+0.18
		Post 40	6.48	1.63		
	MICE	Pre	8.76	3.02	+0.74 *	+0.20
		Post 0	6.81	2.16	+0.95 *	+0.47 *
		Post 20	6.43	1.69	+1.20 *	+0.31
		Post 40	5.95	1.40		
	SED	Pre	8.05	2.50	+0.66 *	−0.10
		Post 0	6.57	1.94	+0.57 *	+0.02
		Post 20	6.76	2.00	+0.64 *	+0.11
		Post 40	6.52	2.25		

Note: Direction of effect size indicates positive (i.e., +) or negative (i.e., −) change in affective state. * indicates significant change at the *p* = 0.05 level.

## Data Availability

Data will be made available upon reasonable request from the first author.
